# Inhibition of Rac1 GTPase Decreases Vascular Oxidative Stress, Improves Endothelial Function, and Attenuates Atherosclerosis Development in Mice

**DOI:** 10.3389/fcvm.2021.680775

**Published:** 2021-08-06

**Authors:** Sebastian Zimmer, Philip Roger Goody, Matthias Oelze, Alexander Ghanem, Cornelius F. Mueller, Ulrich Laufs, Andreas Daiber, Felix Jansen, Georg Nickenig, Sven Wassmann

**Affiliations:** ^1^Department of Internal Medicine II, Heart Center Bonn, University Hospital Bonn, Bonn, Germany; ^2^Zentrum für Kardiologie - Kardiologie I, Universitätsmedizin der Johannes Gutenberg-Universität, Mainz, Germany; ^3^Department of Internal Medicine II - Cardiology and Medical Intensive Care, Asklepius Hospital Nord - Heidberg, Hamburg, Germany; ^4^Department of Cardiology, University Hospital Leipzig, Leipzig, Germany; ^5^Cardiology Pasing, Munich, Germany; ^6^Department of Inernal Medicine III, Saarlang University Medical Center, Homburg, Germany

**Keywords:** atherosclerosis, endothelial function, oxidative stress, free radicals, Rac1, GTPases

## Abstract

**Aims:** Oxidative stress and inflammation contribute to atherogenesis. Rac1 GTPase regulates pro-oxidant NADPH oxidase activity, reactive oxygen species (ROS) formation, actin cytoskeleton organization and monocyte adhesion. We investigated the vascular effects of pharmacological inhibition of Rac1 GTPase in mice.

**Methods and Results:** We treated wild-type and apolipoprotein E-deficient (ApoE^−/−^) mice with *Clostridium sordellii* lethal toxin (LT), a Rac1 inhibitor, and assessed vascular oxidative stress, expression and activity of involved proteins, endothelial function, macrophage infiltration, and atherosclerosis development. LT-treated wild-type mice displayed decreased vascular NADPH oxidase activity and ROS production. Therapeutic LT doses had no impact on behavior, food intake, body weight, heart rate, blood pressure, vascular and myocardial function, differential blood count, and vascular permeability. ApoE^−/−^ mice were fed a cholesterol-rich diet and were treated with LT or vehicle. LT treatment led to decreased aortic Rac1 GTPase activity, NADPH oxidase activity and ROS production, but had no impact on expression and membrane translocation of NADPH oxidase subunits and RhoA GTPase activity. LT-treated mice showed improved aortic endothelium-dependent vasodilation, attenuated atherosclerotic lesion formation and reduced macrophage infiltration of atherosclerotic plaques. Concomitant treatment of cholesterol-fed ApoE^−/−^ mice with LT, the specific synthetic Rac1 inhibitor NSC 23766 or simvastatin comparably reduced aortic Rac1 activity, NADPH oxidase activity, oxidative stress, endothelial dysfunction, atherosclerosis development, and macrophage infiltration.

**Conclusions:** These findings identify an important role of the small GTPase Rac1 in atherogenesis and provide a potential target for anti-atherosclerotic therapy.

## Introduction

Reactive oxygen species (ROS) are thought to be involved in the pathogenesis of atherosclerosis because pathological conditions such as hypertension, hypercholesterolemia and diabetes are associated with an increase in vascular ROS production and predispose for cardiovascular events. In fact, increased levels of ROS have been demonstrated in all layers of the diseased arterial wall and within atherosclerotic plaques. Furthermore, there is ample evidence that oxidative stress is directly involved in the pathogenesis of endothelial dysfunction and ultimately atherosclerosis development by numerous molecular and cellular mechanisms (1–6).

Under physiological conditions, ROS formation and elimination are delicately balanced in the vascular wall. Enhanced activity of pro-oxidant enzymes and/or reduced activity of anti-oxidant enzymes, however, lead to oxidative stress. A major source of ROS in vascular cells is NADPH oxidase and this is recognized to be involved in vascular pathology (7). This enzyme consists of two membrane-bound subunits p22-phox and Nox1 or Nox2, two cytosolic subunits p67-phox and p47-phox and the small GTPase Rac1. Nox4 is another subunit that is present in vascular cells (8–11). These subunits assemble on activation to form the functional enzyme, which then catalyses an electron transfer to molecular oxygen producing superoxide radicals.

NADPH oxidase is activated by many important modulators of vascular cell activity (12) including endothelin-1 (11), vascular endothelial growth factor (13), transforming growth factor-β (14), angiotensin II (15), and mechanical stimuli (16, 17). The small GTPase Rac1 plays a central common role in activation of NADPH oxidase. It interacts with other subunits and tethers the complex of cytosolic subunits to the cell membrane (18, 19). Expression of a dominant negative Rac1 allele/mutant in human aortic endothelial cells and vascular smooth muscle cells, respectively, abolishes ROS production by the NADPH oxidase (20, 21). Rac1 expression and activity is further stimulated by angiotensin II, an effect that can be prevented by 3-hydroxy-3-methylglutaryl coenzyme A reductase inhibitors (e.g., statins) that inhibit geranylgeranylation of the GTPase (22). In addition, ROS production in response to statin withdrawal appears to be caused by enhanced activation and anchoring of Rac1 to the plasma membrane (23).

Rac1 GTPase, a member of the rho family of small GTPases, also plays an important role in organization of the actin cytoskeleton. Actin modulates cell shape, cell-cell contacts and interaction with adhesion molecules. Cellular motility depends on a rapid response to dynamic signals, and ROS have emerged as key mediators. All Rho family members, but especially Rac1 regulate actin cytoskeleton dynamics (24, 25). It has been shown that Rac1 is involved in monocyte adhesion to the endothelium by modulation of the actin cytoskeleton but not ROS release (26).

Rac1 GTPase can be specifically inhibited by *Clostridium sordellii* lethal toxin (LT). LT is a glucosyltransferase that selectively uses UDP-glucose as a co-substrate to glucosylate Rac1 (27). LT effectively inhibits Rac1 in vascular cells. We have previously demonstrated that increased ROS production in vascular smooth muscle cells after stimulation of Rac1 by angiotensin II can be completely inhibited by LT (22).

We hypothesized that *in vivo* inhibition of Rac1 GTPase results in reduced NADPH oxidase activity and thus diminished ROS production in the arterial wall, and that chronic Rac1 inhibition in a mouse model of atherosclerosis will improve endothelial function and reduce atherosclerosis development and macrophage infiltration in atherosclerotic lesions. In the present work we have used specific inhibitors of Rac1 activity to investigate the role of the GTPase in atherosclerosis using a mouse model and conclude that Rac1 inhibition could indeed be an important therapeutic principle.

## Methods

The Methods section of this manuscript can be found in the Supplementary Material.

## Results

### Proof of Concept and Dose Finding Study

The first experiment was designed as a proof of concept and dose finding study. To determine the highest tolerated dose of LT with a relevant effect on vascular ROS production, we treated 12-week-old wild-type mice with 0.1 or 1.0 μg LT/week continuously via osmotic mini-pumps for 7 days. We then measured vascular ROS production and NADPH oxidase activity. Mice that received vehicle or 0.1 μg LT/week behaved normally and had a sustained food and water consumption. Treatment with this lower dose of LT resulted in decreased vascular ROS production (100 ± 29% vs. 49 ± 25%, *n* = 3 per group) and significantly reduced NADPH oxidase activity (100 ± 19% vs. 47 ± 7%, *p* < 0.05 vs. vehicle, *n* = 3 per group) compared to controls. Mice treated with the higher dose showed no external signs of illness but were apathetic and had a reduced food and water intake. Therefore, 0.1 μg LT/week was used in all of the following experiments.

### Short-Term Rac1 Inhibition and Endothelial Function

To investigate whether Rac1 GTPase inhibition with LT and the associated reduction in vascular oxidative stress affects endothelial dysfunction in mice, we first studied endothelial function in atherosclerotic mice treated with LT. ApoE^−/−^ mice were fed a cholesterol-rich, high-fat diet for 7 weeks and were treated with 0.1 μg LT/week or vehicle during the last 7 days of diet. Wild-type mice that received high-fat, cholesterol-rich diet showed normal vasodilation in intact aortic ring preparations. ApoE^−/−^ mice developed severely impaired endothelium-dependent vasodilation, indicating endothelial dysfunction. Rac1 GTPase inhibition with LT significantly improved endothelium-dependent vasodilation in these ApoE^−/−^ mice (maximal relaxation: wild-type + vehicle 81 ± 7%, ApoE^−/−^ + vehicle 16 ± 2%, ApoE^−/−^ + LT 48 ± 11%, *p* < 0.05 vs. ApoE+vehicle, *p* < 0.05 vs. wild-type, *n* = 5 per group). Endothelium-independent vasorelaxation was not affected by LT treatment (maximal relaxation: wild-type + vehicle 122 ± 10%, ApoE^−/−^ + vehicle 116 ± 12%, ApoE^−/−^ + LT 117 ± 15%, *n* = 5 per group), neither was KCl- or phenylephrine-induced vasoconstriction (*n* = 5 per group, data not shown).

### Assessment of LT Toxicity

**Figure 1:**
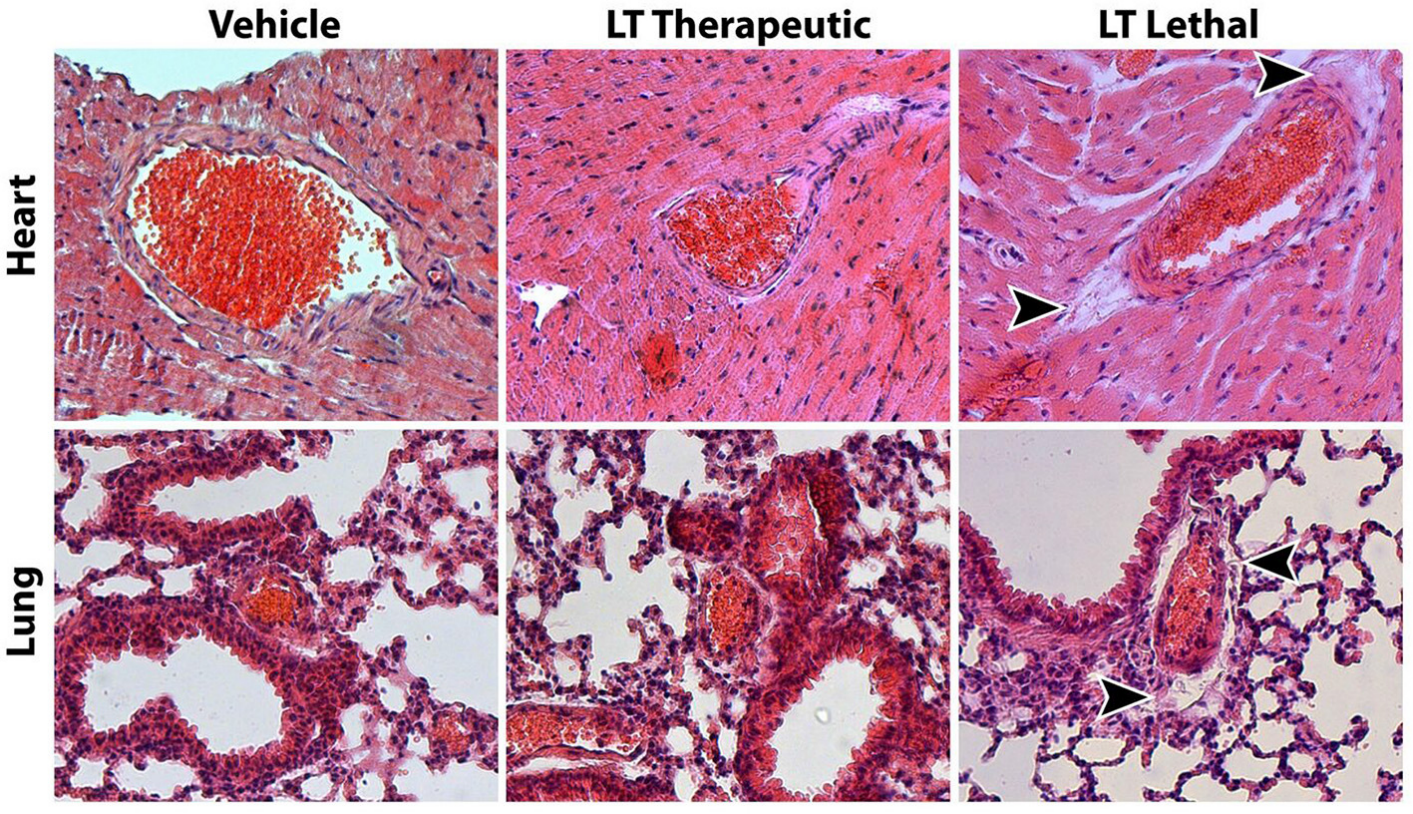
Assessment of *Clostridium sordellii* lethal toxin (LT) toxicity. The main characteristic of LT toxicity is increased vascular permeability with perivascular edema. Representative HE stainings of heart and lung tissue from mice treated with vehicle or a therapeutic dose of LT for 4 weeks show no signs of tissue edema. In contrast, mice that received a lethal dose of LT display edema in the connective tissue surrounding larger arteries (arrow heads).

To significantly influence atherosclerotic plaque development, longer treatments with LT were required. Because high doses of LT lead to increased vascular permeability, tissue edema and death in mice (28), we first investigated the potential toxic effects of long-term, therapeutic-dose LT treatment. Wild-type mice were treated with either vehicle or 0.1 μg LT/week for 4 weeks via osmotic mini-pumps. Consistent with the previous experiments, long-term LT treatment resulted in a significant reduction of vascular ROS production and NADPH oxidase activity (*n* = 5 per group, data not show). The mice had no external signs of acute or chronic illness. Behavior, body weight as well as food and water consumption remained unchanged in both groups. There were no signs of cardiovascular toxicity; specifically blood pressure, heart rate, cardiac and endothelial function were not altered by LT treatment (Table 1). Histological analysis of the heart did not reveal apparent changes (Figure 1). Indicators of increased vascular permeability and tissue edema, including serum albumin concentration and wet/dry ratio of the heart and lung, did not differ between the groups (Table 1). Both vehicle and LT-treated mice had similar differential leukocyte blood counts without evidence of an inflammatory process (Table 1). In contrast, when wild-type mice received a lethal dose of LT (150 ng/kg i.p.), they were soon apathetic, died within 12 h, had significantly lower serum albumin concentrations, altered differential leukocyte blood counts and higher wet/dry ratios of the heart and lung (Table 1). Another sign of LT toxicity is perivascular edema (28). Histological analysis of heart and lung tissues showed perivascular edema exclusively in mice that received a lethal but not therapeutic dose of LT (Figure 1). These results demonstrate that long-term, therapeutic-dose LT treatment does not exert toxicity in our mouse model.

**Table 1:** Assessment of *Clostridium sordellii* lethal toxin (LT) toxicity.

		**Vehicle**	**LT**	**LT lethal**
	Body weight (g)	25.4 ± 0.5	26.1 ± 0.8	25.3 ± 0.8
	Serum albumin (g/l)	18.9 ± 1.3	16.9 ± 1.2	11.3 ± 2.1*
Blood pressure	Systolic (mmHg)	121.5 ± 6.8	122.6 ± 4.7	n.a.
	Diastolic (mmHg)	81.3 ± 7.0	81.8 ± 3.9	n.a.
	Heart rate (bpm)	733 ± 36	740 ± 29	n.a.
Blood count	Lymphocyte (%)	85 ± 5.6	88.2 ± 3.2	53 ± 5.7*
	Neutrophil (%)	8.2 ± 3.8	6.8 ± 0.8	15.5 ± 0.7*
	Monocyte (%)	3.4 ± 1.7	3.2 ± 1.6	31.5 ± 6.4*
	Eosinophil (%)	1.6 ± 1.1	0.6 ± 0.9	0 ± 0
	Basophil (%)	1.8 ± 1.3	1.2 ± 1.3	0.5 ± 0.7
Wet/Dry ratio	Lung	4.2 ± 0.3	4.2 ± 0.24	4.48 ± 0.1*
	Heart	4.08 ± 0.13	4.06 ± 0.36	4.42 ± 0.18*
Echocardiography	LVEDV (μl)	43.7 ± 6.9	43.7 ± 3.1	n.a.
	SV (μl)	35.4 ± 2.1	38.9 ± 2.1	n.a.
	LVM (mg)	133.2 ± 30.7	142.3 ± 26.3	n.a.
	EF (%)	82.3 ± 11.6	82.5 ± 13.7	n.a.
Endothelial function	LogIC50 (M Carbachol)	−5.2 ± 0.33	−5.25 ± 0.14	n.a.
	LogIC50 (M Nitro)	−5.36 ± 0.38	−5.43 ± 0.51	n.a.

*Wild-type mice were treated for 4 weeks with vehicle or LT in the therapeutic dose of 0.1 μg LT/week via osmotic mini-pumps or received a lethal dose of LT (150 ng/kg i.p.), and body weight, heart rate, blood pressure, endothelial function, echocardiographic parameters, differential blood count and markers of vascular permeability were evaluated. LVEDV, left ventricular end-diastolic volume; SV, stroke volume; LVM, left ventricular mass; EF, ejection fraction*.

* *p < 0.05*.

### Long-Term Rac1 Inhibition, Oxidative Stress, and Atherosclerosis

**Figure 2:**
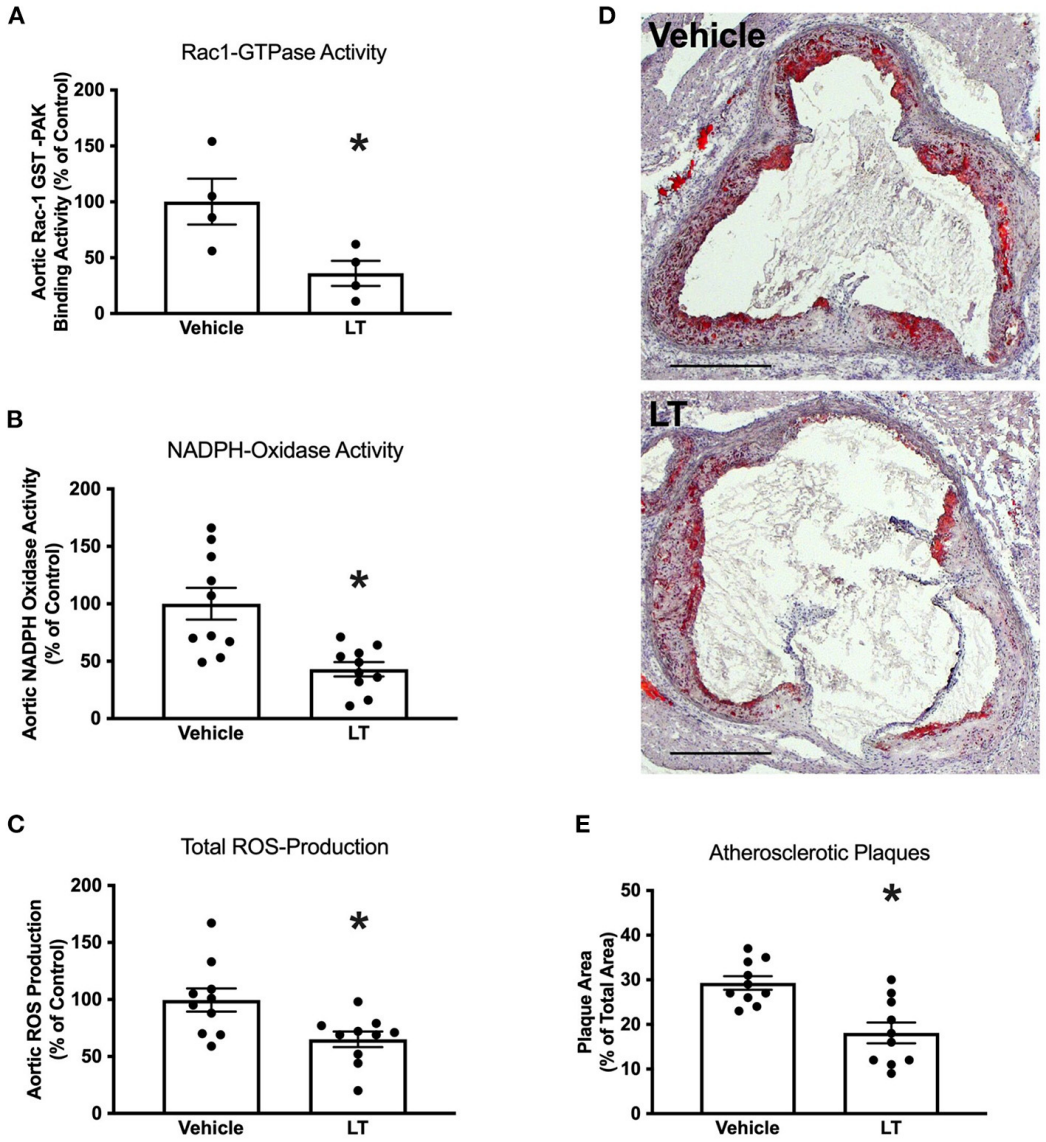
Long-term Rac1 inhibition, oxidative stress and atherosclerosis. **(A)** Aortic homogenates of ApoE^−/−^ mice that were fed a cholesterol-rich diet for 7 weeks and were concomitantly treated with 0.1 μg LT/week via osmotic mini-pumps showed reduced Rac1 GTPase activity compared to vehicle-treated mice in a Rac1 GST-PAK pull-down assay (*p < 0.05, *n* = 4 per group). **(B,C)** LT-treated ApoE^−/−^ mice displayed reduced aortic NADPH oxidase activity (**B**, *p < 0.05, *n* = 10 per group) and aortic ROS production (**C**, *p < 0.05, *n* = 10 per group), as measured by lucigenin-enhanced chemiluminescence and L-012 chemiluminescence, respectively. **(D)** Representative histological cross-sections of the aortic root stained with oil red O to display atherosclerotic plaques. LT-treated ApoE^−/−^ mice showed decreased atherosclerotic plaque formation. **(E)** Quantification of atherosclerotic plaque formation, expressed as plaque area in percent of total area (*p < 0.05, *n* = 10 per group). Scale bars indicate 0.5 mm.

Next, the effect of long-term Rac1 GTPase inhibition by LT on atherosclerosis development was tested. Twelve week-old ApoE^−/−^ mice were fed a high-fat, cholesterol-rich diet and were concomitantly treated with 0.1 μg LT/week or vehicle for 7 weeks. Aortic Rac1 GTPase activity was significantly reduced after treatment with LT (100 ± 22% vs. 36 ± 13%, *p* < 0.05 vs. vehicle, *n* = 4 per group; Figure 2A). Consistently, aortic NADPH oxidase activity (100 ± 20% vs. 43 ± 10%, *p* < 0.05 vs. vehicle, *n* = 10 per group; Figure 2B) and aortic ROS production (100 ± 13% vs. 65 ± 5%, *p* < 0.05 vs. vehicle, *n* = 10 per group; Figure 2C) were significantly decreased in the LT-treated animal group. To investigate whether Rac1 inhibition in the aortic wall affects atherosclerosis, we assessed atherosclerotic plaque formation in the aortic root of these mice. Figures 2D,E reveal that atherosclerotic lesion formation was significantly reduced in ApoE^−/−^ mice treated with LT compared to vehicle-treated animals (29 ± 2% vs. 18 ± 4%, *p* < 0.05 vs. vehicle, *n* = 10 per group). To validate that the observed effects were not mediated by a change in cholesterol levels, we measured total plasma cholesterol concentrations after the 7-week high-fat, cholesterol-rich diet. There was no significant difference between ApoE^−/−^ mice that received vehicle and those treated with LT (1,318 ± 46 mg/dl vs. 1,414 ± 51 mg/dl, *n* = 10 per group).

### NADPH Oxidase Subunit Expression

**Figure 3:**
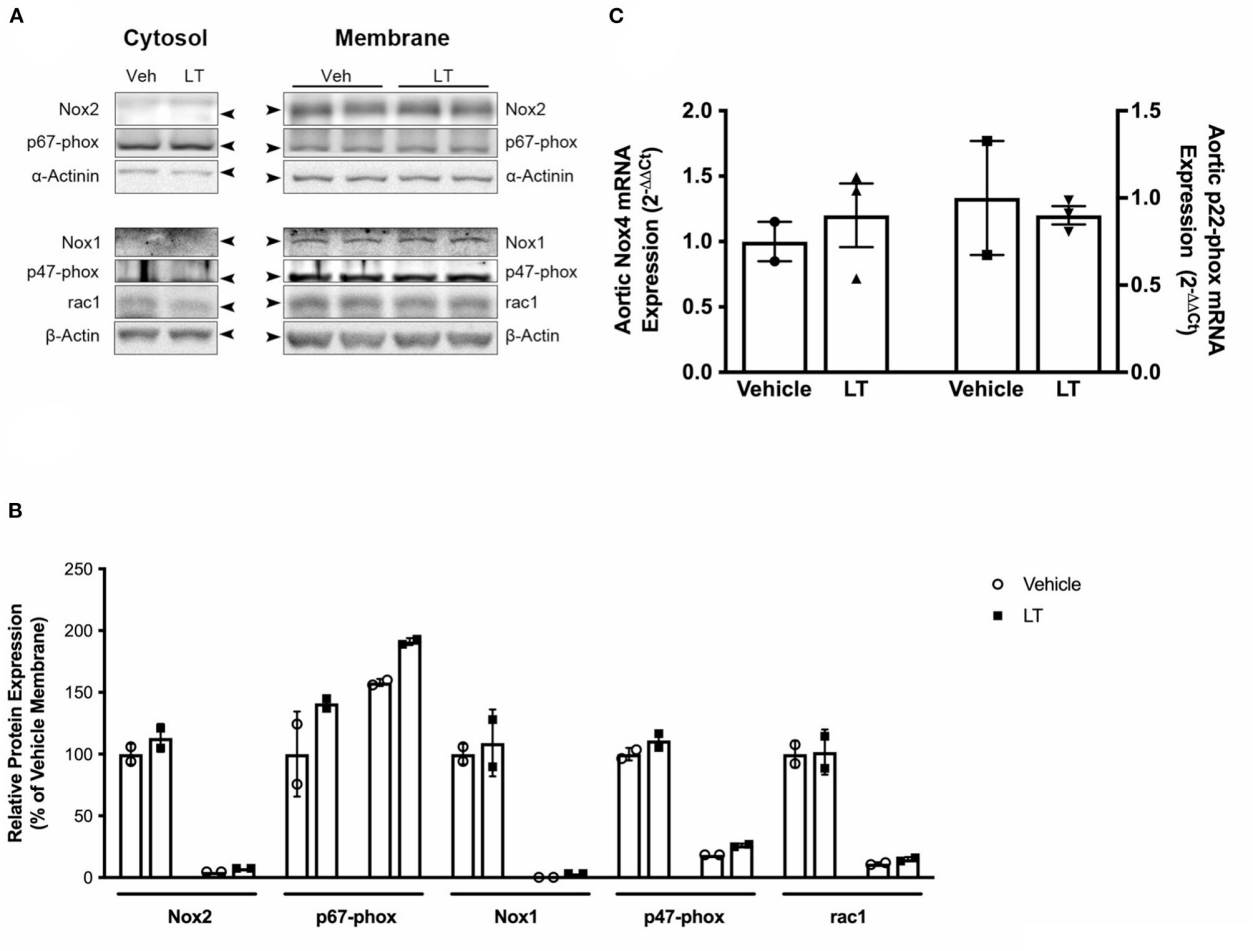
NADPH oxidase subunit expression. **(A)** Western blot analysis of NADPH oxidase subunit protein expression in cytosolic and membrane fractions of aortic homogenates of cholesterol-fed ApoE^−/−^ mice treated with vehicle or LT. There was no difference in Rac1, Nox1, Nox2, p47-phox, and p67-phox expression. **(B)** Densitometric quantification of the Western analyses. Expression of genes-of-interest was normalized to housekeeping gene expression. These values were normalized to membrane protein expression of vehicle-treated mice (*n* = 2 per group). For each subunit, membrane expression is displayed on the left, cytosolic expression on the right. **(C)** Real-time PCR analysis of Nox4 and p22-phox subunit mRNA expression in aortic homogenates of cholesterol-fed ApoE^−/−^ mice treated with vehicle or LT. Neither aortic Nox4 nor p22-phox expression was altered by LT treatment in these mice (*n* = 2–3 independent measurements (pooled from n=2-3 mice per sample) per group).

To assess whether LT in addition to inhibition of Rac1 GTPase activity also influences NADPH oxidase expression, NADPH oxidase subunits were analyzed in aortic tissue of the above-mentioned ApoE^−/−^ mice. There were no significant differences in protein expression levels of the NADPH oxidase subunits Rac1, p47-phox, p67-phox, Nox1, and Nox2 between the groups (Figures 3A,B). Furthermore, there was no difference in Rac1, p47-phox, or p67-phox expression in cytosolic and membrane protein fractions, indicating that there were no changes in membrane translocation of these cytosolic subunits (Figures 3A,B). Moreover, neither Nox4 (1.0 ± 0.15 2^−ΔΔCt^ vs. 1.2 ± 0.24 2^−ΔΔCt^, *n* = 2–3 per group; Figure 3C left) nor p22-phox (1.0 ± 0.32 2^−ΔΔCt^ vs. 0.9 ± 0.05 2^−ΔΔCt^, *n* = 5 per group; Figure 3C right) mRNA expression was altered by LT treatment.

### Comparison of Different Rac1 Inhibitors

**Figure 4:**
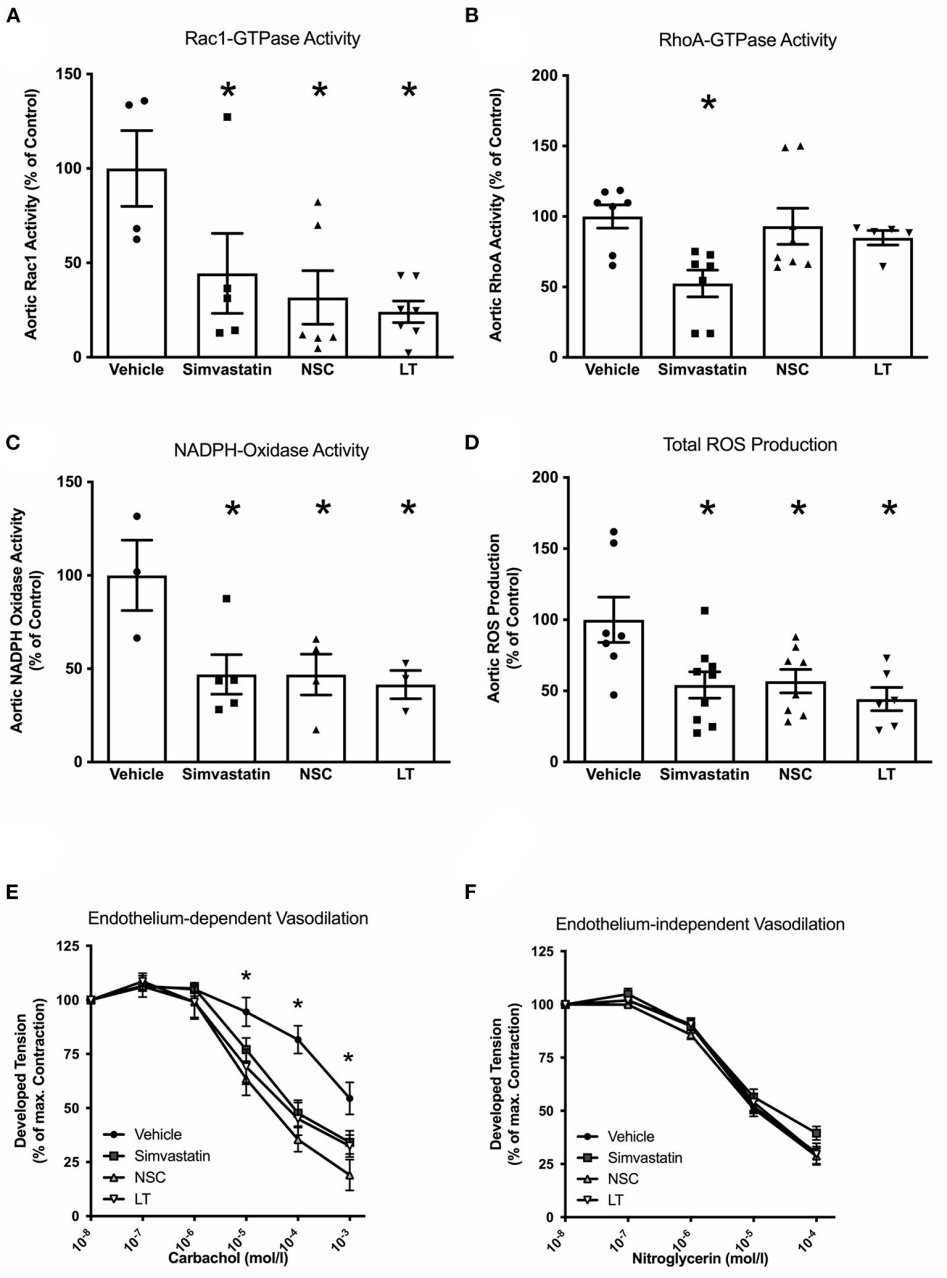
Vascular effects of different Rac1 inhibitors: GTPase activity, oxidative stress and endothelial function. To investigate the vascular effects of different Rac1 GTPase inhibitors, ApoE^−/−^ mice were fed a cholesterol-rich diet for 7 weeks and were concomitantly treated with vehicle, LT (0.1 μg LT/week), NSC 23766 (10 mg/kg/d), or simvastatin (20 mg activated simvastatin/kg/d). All three compounds significantly inhibited aortic Rac1 GTPase activity (**A**, Rac1 GTPase activity G-LISA, *p < 0.05 vs. vehicle, *n* = 4–7 per group), but only simvastatin significantly decreased aortic rhoA GTPase activity (**B**, rhoA GTPase activity G-LISA, *p < 0.05 vs. vehicle, *n* = 5–8 per group). All three compounds significantly reduced aortic NADPH oxidase activity (**C**, lucigenin-enhanced chemiluminescence, *p < 0.05 vs. vehicle, *n* = 3–5 per group) and aortic ROS production (**D**, L-012 chemiluminescence, *p < 0.05 vs. vehicle, *n* = 6–9 per group) and significantly improved endothelium-dependent vasodilation (**E**, organ chamber experiments with isolated aortic segments, *p < 0.05 vs. vehicle, *n* = 5 per group) compared to vehicle-controls. Endothelium-independent vasodilation was not affected by the Rac1 inhibitors (**F**, *n* = 5 per group).

**Figure 5:**
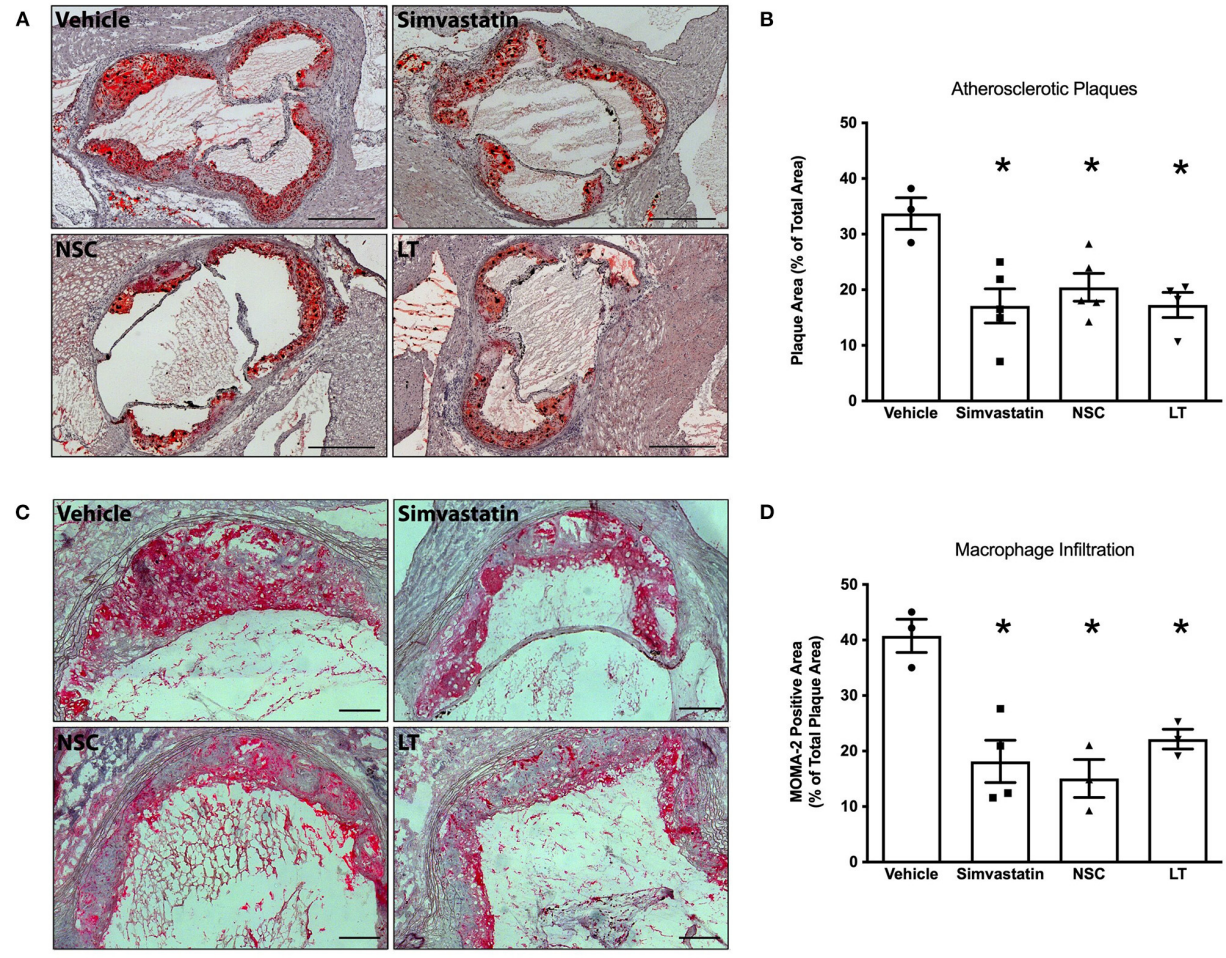
Vascular effects of different Rac1 inhibitors: atherosclerosis and macrophage infiltration. ApoE^−/−^ mice were fed a cholesterol-rich diet for 7 weeks and were concomitantly treated with vehicle, LT (0.1 μg LT/week), NSC 23766 (10 mg/kg/d), or simvastatin (20 mg activated simvastatin/kg/d). All three compounds significantly reduced atherosclerotic plaque formation in the aortic root compared to vehicle-control. **(A)** Representative histological cross-sections. Scale bars indicate 0.5 mm. **(B)** Quantification of atherosclerotic plaque formation, expressed as plaque area in percent of total area (*p < 0.05 vs. vehicle, *n* = 3–5 per group). Furthermore, all three compounds significantly reduced macrophage infiltration of atherosclerotic plaques in these mice. **(C)** Representative MOMA-2 immunohistochemical staining using a monoclonal rat anti-mouse MOMA-2 antibody. Scale bars indicate 0.15 mm. **(D)** Quantification of macrophages infiltrating aortic atherosclerotic plaques (*p < 0.05 vs. vehicle, *n* = 3–4 per group).

Statins have been shown to reduce Rac1 GTPase activity *in vitro* and *in vivo* (22, 29), and a specific synthetic small-molecule Rac1 GTPase inhibitor, NSC 23766, has recently been developed (30, 31). To test whether these Rac1 inhibitors have comparable vascular *in vivo* effects as LT, ApoE^−/−^ mice were fed a high-fat, cholesterol-rich diet for 7 weeks and were concomitantly treated with vehicle, simvastatin, NSC 23766 or LT. Serum lipoprotein profiles have been previously investigated and no significant effects were measured (32). All three treatments significantly inhibited aortic Rac1 GTPase activity (vehicle 100 ± 21%, simvastatin 44 ± 21%, NSC 32 ± 14%, LT 24 ± 6%, all *p* < 0.05 vs. vehicle, *n* = 4–7 per group; Figure 4A). There have been reports that LT can glucosylate other members of the Rho family of small GTPases to some degree, as for example RhoA. However, only simvastatin but not LT or NSC 23766 significantly inhibited aortic RhoA GTPase activity in the ApoE^−/−^ mice (vehicle 100 ± 8%, simvastatin 53 ± 9%, *p* < 0.05 vs. vehicle, NSC 93 ± 13%, LT 81 ± 6%, *n* = 5–8 per group; Figure 4B). All three compounds significantly reduced aortic NADPH oxidase activity (vehicle 100 ± 19%, simvastatin 47 ± 11%, NSC 47 ± 12%, LT 42 ± 8%, all *p* < 0.05 vs. vehicle, *n* = 3–5 per group; Figure 4C) and aortic ROS production (vehicle 100 ± 16%, simvastatin 54 ± 9%, NSC 57 ± 8%, LT 44 ± 8%, all *p* < 0.05 vs. vehicle, *n* = 6–9 per group; Figure 4D). Treatment with these Rac1 GTPase inhibitors improved endothelium-dependent vasodilation (maximal relaxation: vehicle 46 ± 8%, simvastatin 66 ± 5%, NSC 81 ± 7%, LT 68 ± 5%, all *p* < 0.05 vs. vehicle, *n* = 5 per group; Figure 4E) but had no effect on endothelium-independent vasorelaxation (Figure 4F) or phenylephrine-induced vasoconstriction (data not shown). Importantly, treatment with simvastatin, NSC 23766 or LT significantly attenuated atherosclerotic plaque development in ApoE^−/−^ mice to a similar degree (vehicle 34 ± 3%, simvastatin 17 ± 3%, NSC 20 ± 3%, LT 17 ± 2%, all *p* < 0.05 vs. vehicle, *n* = 3–5 per group; Figures 5A,B). Because Rac1 inhibition diminishes monocyte adhesion to endothelial cells *in vitro*, we studied macrophage infiltration of atherosclerotic plaques in these ApoE^−/−^ mice. Simvastatin, NSC 23766 and LT treatment significantly reduced macrophage infiltration of atherosclerotic plaques to a similar degree compared to vehicle-treated mice (vehicle 41 ± 3%, simvastatin 29 ± 5%, NSC 15 ± 3%, LT 22 ± 2%, all *p* < 0.05 vs. vehicle, *n* = 3–4 per group; Figures 5C,D).

## Discussion

The data in this study demonstrate that long-term inhibition of Rac1 GTPase exerts atheroprotective effects in hypercholesterolemic ApoE^−/−^ mice. Systemic application of *Clostridium sordellii* lethal toxin or the specific synthetic small-molecule inhibitor NSC 23766 effectively inhibit Rac1 GTPase activity in the aorta which in turn significantly reduces NADPH oxidase activity and results in decreased oxidative stress in the vessel wall. Long-term Rac1 inhibition is ultimately associated with improved endothelial function, reduced atherosclerotic plaque formation and diminished macrophage infiltration.

Intraperitoneal injection of high doses of LT in mice induces symptoms similar to fatal toxic shock syndrome observed in humans and animals after bacterial infection, which is mediated by a severe increase in vascular permeability (28, 33, 34). We tested two dosing regimens of LT and found that the lower was well-tolerated by mice, yet it was sufficient to significantly inhibit Rac1 GTPase activity in the aortic wall. In contrast to treatment with a lethal dose of LT, which caused severe pulmonary and cardiac edema, this therapeutic dose of LT had no impact on behavior, food intake, body weight, heart rate, blood pressure, vascular and myocardial function, differential blood count and vascular permeability. It is therefore possible to treat mice systemically with LT over long durations and investigate the effects of chronic Rac1 inhibition without inducing acute illness.

LT is an established model of specific inhibition of Rac1 GTPase activity, and the toxin is highly effective in vascular cells. However, there have been reports that LT can glucosylate other members of the rho family of small GTPases to some degree, as for example RhoA. RhoA has been shown to play an important regulatory role in vascular cells (24). Our data show that LT profoundly inhibits activation of Rac1 GTPase but does not affect RhoA GTPase activation in aortic tissue of ApoE^−/−^ mice. In addition, treatment of ApoE^−/−^ mice with the specific synthetic small-molecule Rac1 GTPase inhibitor NSC 23766, which does not exert effects on other rho family proteins (30, 31), profoundly inhibited Rac1 GTPase but not RhoA GTPase activity and led to identical results as treatment with LT. Although effects on other Rho family proteins cannot be fully excluded, these findings corroborate the notion that LT acts predominantly on Rac1 GTPase in the vascular system of our animal model.

We have previously shown that overexpression of the constitutively active mutant RacL61 increases ROS release in vascular smooth muscle cells and that Rac1 inhibition via LT or overexpression of the dominant-negative RacN17 mutant decreases ROS production (21, 22). We now demonstrate that LT treatment exerts this effect *in vivo* when applied systemically in mice. Rac1 inhibition causes diminished NADPH oxidase activity in the vessel wall, which in turn leads to reduced ROS formation and thus decreased oxidative stress. LT inhibits Rac1 activity by direct glucosylation of the GTPase. However, to evaluate whether LT has additional effects on the NADPH oxidase complex, we investigated NADPH oxidase subunit expression and membrane translocation in aortic homogenates of vehicle or LT-treated ApoE^−/−^ mice. There was no significant difference in Nox1, Nox2, Nox4, p22-phox, p47-phox, p67-phox, and Rac1 subunit expression. Moreover, comparison of Rac1, p47-phox, and p67-phox expression in cytosolic and membrane protein fractions revealed that there were no changes in membrane translocation of these subunits. Inhibition of NADPH oxidase activity through specific inhibition of Rac1 GTPase is a principle mechanism of action underlying the LT-mediated reduction of vascular oxidative stress in our animal model. Although effects on other pro- and antioxidant systems cannot be excluded, the above notion is further supported by our data derived from specific pharmacological Rac1 inhibition with NSC 23766.

Oxidative stress has been implicated in the pathogenesis of atherosclerosis (35). All common risk factors associated with coronary artery disease such as elevated LDL cholesterol, diabetes, hypertension, and cigarette smoking are associated with increased ROS levels within the arterial wall. In fact, it has been demonstrated that increased oxidative stress leads to endothelial dysfunction by countering the vasoprotective and vasodilating effects of nitric oxide (36, 37). It was speculated that decreasing the oxidative stress burden would reduce cardiovascular events but clinical trials with antioxidant therapies failed to show reductions in event rates (38). The lack of benefit seen in these clinical trials does not, however, disprove the central role of oxidative stress in atherosclerosis, but rather implies that the antioxidative agents used were not capable of significantly modifying oxidative stress at the appropriate site and time. Instead of scavenging an excess of ROS, primarily reducing the formation of ROS may be a more efficient approach to decrease vascular oxidative stress and therefore impact on vascular pathologies. Our data suggest that inhibiting a major source of ROS in the vessel wall, NADPH oxidase, is an effective method to decrease vascular oxidative stress and improve endothelial function in an established animal model of atherosclerosis. Endothelial dysfunction is not only an early and crucial stage of atherosclerosis development, but is also associated with increased risk for cardiovascular events (39). ApoE ^−/−^ mice develop atherosclerotic lesions in response to a cholesterol-rich diet and are widely used to study the pathogenesis of atherosclerosis (40). Typically, atherosclerotic lesions are more pronounced in the aortic root and the proximal aorta than in more distal parts (41). Importantly, we provide evidence that long-term Rac1 inhibition significantly reduces atherosclerotic plaque formation in this animal model. Our finding is in agreement with other studies demonstrating diminished atherosclerosis on genetic inhibition of NADPH oxidase and reduction of vascular oxidative stress (42).

Rac1 GTPase not only exerts effects on ROS generation but also on organization of the actin cytoskeleton, which is, among others, important for monocyte adhesion (24). Monocyte adhesion to the endothelium, migration, accumulation, and activation of monocytes in atherosclerotic lesions is a central part of the inflammatory process involved in atherogenesis (39). We have previously demonstrated that Rac1 mediates actin rearrangement, lamellipodia formation and adhesion of human monocytes *in vitro* independently of ROS (26). We now provide evidence of reduced macrophage infiltration of atherosclerotic plaques after long-term Rac1 inhibition, further supporting the importance of Rac1 GTPase for monocyte function *in vivo* and development of atherosclerosis. It should be noted that a further contribution of Rac1 to atherogenesis in the vascular endothelium is by coupling of Rac1 activation to LDL transport across endothelial monolayers via SR-B1 (scavenger receptor, class B type 1) (43). Physiological or chronic activation of Rac1 is achieved through an exchange of guanosine diphosphate (GDP) to guanosine triphosphate, which is catalyzed by the exchange factor Vav2. Vav2 is phosphorylated and thus activated by activated vascular endothelial growth factor (VEGF). Phosphorylation of Vav2 seems to be regulated by Src and Src kinase activity is required for activation of Rac1 (44). Several other Rac1 GEFs, including Tiam or DOCK proteins, could also be important (43, 45).

The findings of our study help to identify Rac1 GTPase as an important protagonist in atherogenesis. Inhibition of Rac1 GTPase by either *Clostridium sordellii* lethal toxin or the specific small-molecule inhibitor NSC 23766 is associated with reduced vascular NADPH oxidase activity and oxidative stress, decreased macrophage infiltration, improved endothelial function and ultimately diminished atherosclerosis development. In our animal model, the vascular and ultimately atheroprotective actions of LT and NSC 23766 were as efficacious as those of simvastatin. This is important because the specific Rac1 inhibitors LT and NSC 23766 do not display lipid-lowering properties or other pleiotropic effects attributed to statins. Additionally, the mode of action of statins on Rac1 is different and involves prevention of geranylgeranylation of Rac1 at its C-terminus. This is expected to prevent membrane association of Rac1, a prerequisite for its activation of the NADPH oxidase. Statins also activate Rac1 via induction of AMP-activated protein kinase (AMPK) and liver kinase B1 (LKB1) phosphorylation (45). However, statins were also shown to inhibit NADPH oxidase activity in a Rac1 independent manner in endothelial cells, which demonstrates that multiple pathways are regulated after statin treatment and might help explain the extent of the observed, and sometimes contradictory, effects (46). Interestingly, prevention of Rac1 prenylation in macrophages by deleting GTPase 1, the prenylase responsible for Rac1 modification, leads to significant inflammatory response in macrophages and to rheumatoid arthritis (46). The authors suggest that this is due to increased activation of unprenylated Rac1 (i.e., generation of the GTP-bound form of the GTPase) and that effector interactions involved in innate immunity are enhanced. This would presumably also apply to statins and recent findings confirm that disruption of Rac1 regulation via statins leads to increased atherosclerotic plaque calcification (47). However, these effects would not be induced by the modes of inhibition used in the present study, thus avoiding the potential negative effects of statins.

Although specific Rac1 inhibition with LT or NSC 23766 was not superior to statin treatment, our findings are decisive because they identify Rac1 GTPase as a key molecule in atherogenesis and provide novel proof of concept that specifically inhibiting this molecule is sufficient to diminish the atherosclerotic process in our animal model and therefore may improve vascular health. Thus, Rac-1 GTPase may represent an interesting target for future anti-atherosclerotic drug development. Further detailed *in vitro* investigations are necessary to establish the exact mechanisms of the observed effects upon Rac1 inhibition.

## Data Availability Statement

The datasets generated and/or analyzed during the current study are available from the corresponding author on reasonable request.

## Ethics Statement

The animal study was reviewed and approved by Landesamt für Natur, Umwelt und Verbraucherschutz Nordrhein-Westfalen, Germany Godesberger Allee 136, 53175 Bonn.

## Author Contributions

SZ, GN, and SW conceived and designed the project. SZ, PG, MO, AG, CM, AD, and FJ performed experiments. SZ, PG, MO, AG, CM, UL, AD, FJ, GN, and SW analyzed and interpreted the data. All authors contributed to writing and editing of the manuscript.

## Conflict of Interest

The authors declare that the research was conducted in the absence of any commercial or financial relationships that could be construed as a potential conflict of interest.

## Publisher's Note

All claims expressed in this article are solely those of the authors and do not necessarily represent those of their affiliated organizations, or those of the publisher, the editors and the reviewers. Any product that may be evaluated in this article, or claim that may be made by its manufacturer, is not guaranteed or endorsed by the publisher.
