# Supplementary material for: Inhibition of Rac1 GTPase Decreases Vascular Oxidative Stress, Improves Endothelial Function, and Attenuates Atherosclerosis Development in Mice
Source: Front Cardiovasc Med. 2021 Aug 6;8:680775. doi: 10.3389/fcvm.2021.680775 (PMC8377253; doi:10.3389/fcvm.2021.680775)
Supplement: Supplementary file 1 [file Data_Sheet_1.doc]

**DATA SUPPLEMENT**

**Methods**

*Materials*

Oil red O solution, simvastatin, salts, and other chemicals were purchased from Sigma-Aldrich Chemical. Simvastatin was activated as recommended by Sigma. L-012 was obtained from Wako Chemicals and NSC 23766 from Tocris Bioscience. Purified *Clostridium sordellii* lethal toxin (LT) was produced by K. Aktories (University of Freiburg, Germany).

*Animals and procedures*

Male, 12-week-old C57BL/6J (wild-type) mice and apolipoprotein E-deficient (ApoE-/-) mice (C57BL/6J genetic background; both Charles River, Sulzfeld, Germany) were used for this study. Animals were maintained in a 22°C room with a 12-hour light/dark cycle and received drinking water ad libitum. To permit subcutaneous infusion of vehicle, LT or NSC 23766 at a constant rate, osmotic mini-pumps (Alzet, Cupertino, CA) were implanted subcutaneously via a 1 cm interscapular incision. For insertion of osmotic mini-pumps, all mice were anesthetized with 150 mg/kg body weight ketaminehydrochloride (Ketanest, Pharmacia) and 0.1 mg/kg body weight xylazinehydrochloride (Rompun 2%, Bayer) i.p. LT-treatment followed 5 different protocols. Protocol 1: Wild-type mice were fed normal rodent chow and were treated with 0.1 µg LT/week, 1.0 µg LT/week or vehicle for 7d. Protocol 2: Wild-type mice were fed normal chow and were treated with 0.1 µg LT/week for 4 weeks. Protocol 3: ApoE-/- mice were fed a high-fat, cholesterol-rich diet for 7 weeks that contained 21% fat, 19.5% casein, and 1.25% cholesterol (Ssniff, Soest, Germany) and were treated with 0.1 µg LT/week or vehicle for the last 7d of diet. Protocol 4: ApoE-/- mice were fed the same high-fat, cholesterol-rich diet for 7 weeks and were concomitantly treated with 0.1 µg LT/week or vehicle for 7 weeks. Protocol 5: ApoE-/- mice were fed the same high-fat, cholesterol-rich diet for 7 weeks and were concomitantly treated with 0.1 µg LT/week, 10mg NSC 23766/kg/d (22), 20mg activated simvastatin/kg/d SC or vehicle for 7 weeks. Plasma cholesterol concentrations and serum albumin concentrations were determined by routine chemical methods. Body weights were measured weekly. Arterial blood pressure and heart rate were assessed with a computerized tail-cuff method (CODA 6, Kent Scientific) before and after treatment. The mice were killed after the indicated treatments and tissue samples and blood were collected immediately. All animal experiments were performed in accordance with institutional guidelines and the German animal protection law.

*Measurement of oxidative stress*

ROS release in intact aortic segments was determined by L-012 chemiluminescence, as previously described (23). Aortas were carefully excised and placed in chilled, modified Krebs-HEPES buffer. Connective tissue was removed and aortas were cut into 2 mm segments. Chemiluminescence of aortic segments was assessed in scintillation vials containing Krebs-HEPES buffer with 100 µmol/l L-012 over 15 min in a scintillation counter (Lumat LB 9501, Berthold, Bad Wildbad, Germany) in 1 min intervals. The vessel segments were then dried and dry weight was determined. ROS release is calculated as relative chemiluminescence per mg aortic tissue.

*Measurement of NADPH oxidase activity*

NADPH oxidase activity was measured by a lucigenin-enhanced chemiluminescence assay in buffer B containing phosphate 50 mmol/l (pH 7.0), EGTA 1 mmol/l, protease inhibitors (Complete, Roche), sucrose 150 mmol/l, lucigenin 0.005 mmol/l, and NADPH 0.1 mmol/l. Aortic tissue was mechanically lysed using a glass/teflon potter in ice-cold buffer B lacking lucigenin and substrate. Total protein concentration was adjusted to 1 mg/ml. Aliquots of the protein sample (100 µl) were measured over 10 minutes in quadruplicates using NADPH as substrate in a scintillation counter (Berthold Lumat LB 9501) in 1-minute intervals.

*Rac1 GST-PAK pull-down assay*

A glutathione-S-transferase (GST)-PAK-CD (PAK-CRIB domain) fusion protein (Millipore, Billerica, MA), containing the Rac1 binding region from human PAK1 was used to determine Rac1 activity, as previously described (16). Aortic tissue was mechanically lysed using a glass/teflon potter in ice-cold magnesium-containing lysis buffer (25 mmol/l HEPES, pH 7.5, 150 mmol/l NaCl, 1% Igepal CA-630, 0.25% sodium deoxycholate, 10% glycerol, 25 mmol/l NaF, 10 mmol/l MgCl2, 1 mmol/l EDTA, 1 mmol/l sodium orthovanadate, 10 µg/ml leupeptin, 10 µg/ml aprotinin), and then centrifuged for 5 min at 4°C to spin down cellular debris. Aliquots were taken from the supernatant for protein quantification. Supernatant protein samples were immediately incubated with the GST-PAK fusion protein bound to glutathione-coupled agar beads at 4°C for 60 min. The beads and proteins bound to the fusion protein were washed three times in an excess of lysis buffer, eluted in 2x Laemmli sample buffer (60 mmol/l Tris, pH 6.8, 2% SDS, 10% glycerin, 0.1% bromophenol blue), and then analyzed for bound Rac1 (i.e. active Rac1-GTP) molecules by SDS-PAGE and subsequent Western analysis. Immunoblotting was performed using a mouse monoclonal Rac1 antibody (Upstate). Values were normalized to sample protein concentrations and expression of total Rac1 and tubulin (Santa Cruz).

*Aortic ring preparations and tension recording*

Vasodilation and vasoconstriction of isolated aortic ring preparations was determined in organ baths filled with oxygenated modified Tyrode buffer (37°C), as previously described (23). Adventitial tissue was carefully removed, and 3-mm segments of the thoracic aorta were investigated. A resting tension of 10 mN was maintained throughout the experiment. Drugs were added in increasing concentrations in order to obtain cumulative concentration-response curves: KCl 20 and 40 mmol/l, phenylephrine 1 nmol/l -10 µmol/l, carbachol 10 nmol/l -100 µmol/l (assessment of endothelium-dependent vasodilation after precontraction with phenylephrine), and nitroglycerin 1 nmol/l -10 µmol/l (assessment of endothelium-independent vasodilation after precontraction with phenylephrine). The drug concentration was increased when vasoconstriction or -relaxation was completed. Drugs were washed out before the next substance was added.

*Histological analysis*

Hearts with ascending aortas were embedded in Tissue Tek OCT embedding medium (Miles), snap-frozen, and stored at –80°C. Samples were sectioned on a Leica cryostat (6 µm), starting at the apex and progressing through the aortic valve area into the ascending aorta and the aortic arch, and were placed on slides. For immunohistochemical analysis, cryosections placed on poly-L-lysine (Sigma) coated slides were assessed for the macrophage marker MOMA-2 with an indirect immunoenzymatic method. Slides were incubated with acetone for 30 min at -20°C. Then, PBS-washed slides were preincubated with 10% normal goat serum (Sigma) for 30 min each. The primary antibody (monoclonal rat anti-mouse MOMA-2 antibody, Acris) was applied for 1h at room temperature and thereafter at 4°C overnight. Slides were then incubated with an alkaline phosphatase-conjugated secondary antibody (goat anti-rat IgG, Sigma) for 1h at room temperature. Color reaction was accomplished with FastRed (Sigma) as a chromogenic substrate. Nuclei were counterstained with hematoxylin. Isotype-specific antibodies were used for negative controls. Sections were washed and mounted with Aquatex mounting medium (Sigma) for light microscopic analysis. For the detection of atherosclerotic lesions, aortic cryosections were fixed with 3.7% formaldehyde for 1h, rinsed with deionised water, stained with oil red O working solution (0.5%) for 30 min, and were rinsed again. Hematoxylin staining was performed according to standard protocols. To investigate perivascular and tissue edema, the cardiac apex and right upper pulmonary lobe were fixed in formaldehyde, embedded in paraffin, sectioned with a microtome, and stained with hematoxylin/eosin and Elastica-van-Gieson according to standard protocols. For differential blood counts, blood films were prepared, Pappenheim-stained, and at least 100 leukocytes manually counted/identified. All sections were examined under a Zeiss Axiovert 200M microscope using AxioVision version 4.5.0 software. For quantification of atherosclerotic plaque formation in the aortic root, plaque area and total area of serial histological sections were measured. Atherosclerosis data are expressed as plaque area in percent of total surface area. MOMA-2 data are expressed in percent of total plaque area. The investigators who performed the histological analyses were blinded to the treatment of the respective animal group.

*Echocardiography*

Left ventricular end-diastolic volume (LVEDV), stroke volume (SV), left ventricular mass (LVM) and left ventricular ejection fraction (EF) were measured to assess cardiac function*.* The mice were anesthetized with isoflurane (0.8 vol %) and fur on the chest was depilated with Pilca depilation mousse. Short and long axis images were taken with a ATL HDI 5000 SonoCT XRES using a ATL HDI CL15-7 Entos Probe/Transducer at a heart rate > 400/min. Methods for calculating LVEDV, SV, LVM and EF have been previously described (24, 25).

*Wet/Dry ratio*

The left upper pulmonary lobe and a segment of the heart were collected and weighed immediately after death. The specimen were then dried at 110°C for 24h and weighed again. Wet/dry ratios were calculated for each mouse (26).

*Rac1 and RhoA GTPase activity*

Vascular Rac1 and RhoA GTPase activity was measured using commercially available G-LISA kits (Cytoskeleton Inc.) and in concordance with the manufacturer’s protocols. Briefly, connective tissue was removed from the aortic arch which was then collected and snap frozen in liquid nitrogen. The tissue was homogenized in 400µl ice-cold lysis buffer, and the samples were aliquoted and adjusted to 1.0mg/ml total protein. Sample lysates were then incubated in a GTP-bound Rac1 or RhoA binding 96-well-plate. Next, primary anti-Rac1 or anti-RhoA antibodies were bound to their respective protein and subsequently marked with HRP-bound secondary antibodies. HRP was finally detected using chemiluminometric (Rac1) and colorimetric (RhoA) approaches.

*Protein preparation and Western blotting*

Sample preparation and Western blotting was performed as described previously (27, 28). Briefly, mouse aortic tissue was homogenized in liquid nitrogen. Cytosolic and membrane fractions were obtained by ultracentrifugation (1h, 100,000xg, 4°C) and subjected to SDS-PAGE/electro-blotting on nitrocellulose membranes (BioRad). The blots were incubated with primary antibodies against p67-phox (monoclonal, BD), p47-phox (polyclonal, Upstate), Rac1 (monoclonal, BD), Nox1 (polyclonal, Santa Cruz), Nox2 (monoclonal, BD), α-actinin (monoclonal, Sigma) and β-actin (polyclonal, Sigma), followed by the respective secondary antibodies. Immunodetections were accomplished with either SuperSignal Substrate (Pierce) or ECL Reagent (Amersham). The bands were evaluated by densitometry. P67-phox and Nox2 were normalized to α-actinin expression, whereas Nox1, p47-phox and Rac1 were normalized to β-actin expression.

*RNA isolation and real-time PCR*

For assessment of vascular gene expression, mouse aortas were excised, quickly frozen in liquid nitrogen, and homogenized with a motorized homogenizer. RNA was isolated with peqGOLD RNA-Pure (peqLAB Biotechnology). RNA concentration and quality was verified with a spectrophotometer. Then, 1 µg of the isolated total RNA was reverse transcribed using random primers and MMLV reverse transcriptase (Invitrogen) for 60 minutes at 42°C and 10 minutes at 75°C. The single-stranded cDNA was amplified by real-time quantitative reverse transcription-polymerase chain reaction (RT-PCR) with the TaqMan system (ABI-7500 fast PCR System) using commercially available TaqMan probes (Applied Biosystems) and implemented according to the manufacturer´s protocols. Nox4 and p22-phox mRNA expression was normalized to GAPDH expression (TaqMan probes, Applied Biosystems).

*Statistical analysis*

Data are presented as mean ± standard error of mean (SEM). For statistical analysis, 2-tailed, unpaired Student's t-test and ANOVA for multiple comparisons were employed where applicable. Post-hoc comparisons were performed with the Neuman-Keuls test. p<0.05 indicates statistical significance.
